# Division Spinal Cord in Neck

**Published:** 1874-01

**Authors:** 


Division of the Spinal Cord in the Neck.—(*Indian Medical Gazette,* September 1, 1873.)—N. B. Baillie records the case of a woman who lived for six hours after receiving a blow with a hatchet which cut through the third spinous process and the back part of the fourth cervical vertebra, dividing the spinal cord completely, and penetrating into the body of the vertebra in front of the spinal canal.—*Phil. Med. Times.*

